# *Ochoterenella esslingeri* n. sp. (Nematoda: Onchocercidae: Waltonellinae) from *Bokermannohyla luctuosa* (Anura: Hylidae) in Minas Gerais, Brazil, with notes on *Paraochoterenella* Purnomo & Bangs, 1999

**DOI:** 10.1051/parasite/2012194341

**Published:** 2012-11-15

**Authors:** S. Souza Lima, B. Marun, P.V. Alves, O. Bain

**Affiliations:** 1 Universidade Federal de Juiz de Fora, Departamento de Zoologia, Laboratório de Taxonomia e Ecologia de Helmintos, Campus Universitário, Martelos, CEP 36036-900 Juiz de Fora Minas Gerais Brazil; 2 Universidade Federal do Rio de Janeiro, Instituto de Biofísica Carlos Chagas Filho, Laboratório de Biologia de Helmintos Otto Wuckerer Rio de Janeiro Brazil; 3 Muséum National d’Histoire Naturelle, Parasitologie comparée, UMR 7205 CNRS CP52 61 rue Buffon 75231 Paris Cedex 05 France

**Keywords:** Filarioidea, Waltonellinae, *Ochoterenella*, *Paraochoterenella*, Anura, Hylidae, *Bokermannohyla luctuosa*, Neotropical Realm, Filarioidea, Waltonellinae, *Ochoterenella*, *Paraochoterenella*, Anura, Hylidae, *Bokermannohyla luctuosa*, région néotropicale

## Abstract

The waltonelline *Ochoterenella esslingeri* n. sp., a filarial parasite of the anuran *Bokermannohyla luctuosa* in Minas Gerais, Brazil is described. Several characters distinguish this new species from the 15 species presently included in the genus: the cuticular ornamentation of the female that is restricted to the posterior region of the body, the irregular arrangement of the small, rounded bosses, the postoesophageal vulva, the short glandular oesophagus, the size and shape of the microfilariae, the long left spicule and high spicular ratio. Irregularly arranged, tiny, rounded bosses are common in the monotypic genus *Paraochoterenella* from an Indonesian ranid, which is not well defined but likely valid. In the Neotropical Realm, the type hosts of the species of *Ochoterenella* are Hylidae (*O. esslingeri* n. sp.), Leptodactylidae (two species) and the remaining 13 species were described from the giant toad *Rhinella marina* (Bufonidae).

## Abstract (fr)

Description d’une filaire Waltonellinae *Ochoterenella esslingeri* n. sp., parasite d’un anoure, *Bokermannohyla luctuosa*, au Minas Gerais, Brésil. La nouvelle espèce est distincte des 15 autres du genre par l’ornementation cuticulaire de la femelle limitée à la région postérieure du corps et faite de petites bosses rondes distribuées irrégulièrement, la vulve postoesophagienne, l’oesophage glandulaire court, la taille et la forme de la microfilaire, le spicule gauche long et le rapport spiculaire élevé. Les bosses petites, rondes et distribuées irrégulièrement se retrouvent chez le genre monotypique *Paraochoterenella*, un parasite de ranidé indonésien, dont la définition n’est pas claire, mais qui est vraisemblablement un genre valable. En région néotropicale, les hôtes types des espèces d’*Ochoterenella* sont des Hylidae (*O. esslingeri* n. sp.), Leptodactylidae (deux espèces) et Bufonidae, précisément le crapaud géant *Rhinella marina* (les 13 autres espèces).

## Introduction

The filarial Onchocercidae Leiper, 1911 from anurans belong to either the Waltonellinae Bain & Prod’Hon, 1974 or the Icosiellinae Anderson, 1958. The presence of a long tail and the absence of cephalic spines distinguish the Waltonellinae from the Icosiellinae, which have a very short tail and bear two pairs of submedian cephalic spines (Anderson & Bain, 1976). The present filariae from an anuran host in Brazil belong to the first subfamily.

The Waltonellinae are represented by four genera that were redefined by Esslinger (1986a, b) namely *Foleyellides* Caballero, 1935 (= *Waltonella* Schacher, 1975), *Ochoterenella* Caballero, 1944, *Madochotera* Bain & Brunhes, 1968, *Paramadochotera* Esslinger, 1986, and a fifth, less clearly defined genus, *Paraochoterenella* Purnomo & Bangs, 1999. Currently, *Ochoterenella* is the only genus reported from South and Central America. Several species are known only by the female and microfilariae. The genus is remarkably diverse in a bufonid, the giant toad *Rhinella marina* (Linnaeus, 1758), while only two of a total of 15 species were described from Leptodactylidae. The specimens described here comprise males and females, and possess the main characteristics of *Ochoterenella*. Intersestingly, the type host is a hylid.

## Material And Methods

The filariae were recovered from a single heavily infected specimen of *Bokermannohyla luctuosa* (Pombal & Hadad, 1993), captured in the Municipal Park Lajinha (21º 47’ 45.3’’ S – 43º 22’ 14.9’’ W), Juiz de Fora, state of Minas Gerais, Brazil. Living nematodes could be observed through the host’s skin and the anuran was euthanized. Subsequently, the body cavity was opened by a longitudinal ventral incision from the cloacal opening to the mouth. The filariae were removed from the body cavity and muscular aponeuroses of the thighs. They were fixed in AFA (95 parts 70 % ethanol, three parts 40 % formalin, and two parts glacial acetic acid), stored in 70 % ethanol and cleared in lactophenol for examination. The anterior extremity was studied in apical view, after the head was cut with a razor blade.

In Waltonellinae the cuticular ornamentation is of taxonomic importance (Bain & Prod’Hon, 1974; Esslinger, 1986a, 1989). The presence of cuticular bosses and their arrangement was analyzed, the diameter of these bosses and distances between them were measured at levels defined by Esslinger (1986a): at mid-body of the females and at three times the length of the oesophagus from the apex of the males. The width of the lateral chords in lateral view were measured or illustrated at several levels. Samples of microfilariae were extracted from the uterus near the ovijector for detailed study, and the ovijector was dissected out in one specimen. The ratio of the oesophagus length/body length is given as a percentage, and the vulvar ratio is distance of vulva from anterior extremity/body length, also given as a percentage. The tail ratio is the tail length/body length, expressed as a percentage. The spicular ratio is the length of left/right spicule. Specimens were drawn using a microscope equipped with a camera lucida. Measurements were made on drawings and are given in micrometres, except where otherwise stated. Authority names and dates of the species of *Ochoterenella* are listed in Table III, as well as the type host and family and its geographic origin. The nomenclature of anuran hosts follows that of Frost (2009).

**Table I.** Morphological characteristics of the females of *Ochoterenella esslingeri* n. sp. from *Bokermannohyla luctuosa* in Minas Gerais, Brasil.

Type specimens	Paratype	Holotype	Paratype	Paratype	Paratype	Paratype	Paratype	Mean	SD
Specimen number	1	**2**	3	4	5	6	7	–	–
Body length (mm)	36.6	**34.7**	36.5	35.2	36.5	34.5	37.7	35.9	±1.3
Body width at mid-body	450	**440**	470	390	450	320	440	422.9	± 51.6
Body width at nerve ring	220	**200**	195	200	202	160	212	198.4	± 18.9
Body width at end of muscular oesophagus	260	**230**	210	230	220	200	235	226.4	± 19.3
Body width at level of vulva	500	**485**	490	ND	ND	470	520	493	± 18.6
Cephalic plate: lateral x dorso-ventral	53 x 36	**55 x 30**	58 x 35	ND	ND	ND	ND	55 x 34	ND
Nerve ring to apex	295	**270**	275	210	265	250	270	262.1	± 26,6
Oesophagus total length	1360	**1330**	1570	1132	1410	1380	1410	1370	± 130.2
Glandular oesophagus length	1030	**1040**	1250	885	1020	1050	1040	1045	± 106.9
Oesophagus ratio	3.7	**3.8**	4.3	3.2	3.9	4	3.7	3.8	± 0.3
Position of vulva in relation to digestive tract	intestinal	**intestinal**	intestinal	intestinal	intestinal	intestinal	intestinal	–	–
Distance vulva — anterior extremity	193S	**1930**	2360	1672	1900	1770	1810	1911	± 219.8
Vulvar ratio	5.3	**5.6**	6.5	4.8	5.2	5.l	4.8	5.3	± 0.6
Ovijector length	ND	**ND**	ND	2920	ND	ND	ND	–	–
Tail length	250	**200**	260	250	340	265	320	269.3	± 46.9
Tail width at anus	122	**100**	135	100	120	125	170	124.6	± 23.8
Cuticular bosses at mid-body (dorsal and ventral)	absent	**absent**	absent	absent	absent	absent	absent	–	–
Cuticular bosses on tail region (dorsal and ventral)	present	**present**	present	750–130*	present	present	present	–	–
Distance between bosses on tail region	irregular	**irregular**	irregular	irregular	irregular	irregular	irregular	–	–
Diameter of cuticular bosses on ventral/dorsal surface of tail	3/4	**ND**	3/ND	4/5	4/6	4/3	4/ND	ND	ND

* from caudal end; SD: standard deviation; ND: not determined; measurements are in micrometres, unless otherwise stated.

**Table II.** Morphological characteristics of the males of *Ochoterenella esslingeri* n. sp. from *Bokermannohyla luctuosa* in Minas Gerais, Brasil.

Type specimens	Paratype	Allotype	Paratype	Paratype	Mean	SD
Specimen number	1	**2**	3	4	–	–
Body length (mm)	18.8	**19.1**	14.9	17.9	17.7	± 1.9
Width at mid-body	250	**260**	190	280	245	± 38.7
Width at nerve ring	120	**130**	105	150	126.3	± 18.9
Width at oesophago-intestinal junction	220	**260**	190	220	222.5	± 28.7
Cephalic plate: length x width	54 x 30	**ND**	ND	ND	ND	ND
Parastomal structures: height x width	2.5 x 2.5	**3 x 3**	ND	ND	ND	ND
Buccal capsule	8 x 7	**8 x 7**	6 X 8	ND	ND	ND
Nerve ring to apex	250	**230**	190	234	226	± 25.5
Oesophagus total length	1160	**1050**	890	1052	1038	± 96.3
Glandular oesophagus length	840	**790**	640	770	760	± 85.3
Oesophagus ratio	6,2	**5,5**	6	5,9	5,9	0,29
Tail length	155	**160**	160	203	169,5	± 22.5
Tail width at anus	90	**90**	70	118	92	± 19.7
Caudal papillae: -precloacal	1^a^ + 2^b^	**1 + 2**	1 + 2	1 + 2	ND	ND
- postcloacal	2 + 2 + 2	**2 + 2 + 2**	2 + 2 + 2	2 + 2 + 2	ND	ND
Left spicule length	337	**345**	345	320	336.8	± 11.8
Distal extremity of left spicule	pointed membrane	**pointed membrane**	pointed membrane	pointed membrane	–	–
Right spicule length	135	**115**	130	91	117.8	± 19.8
Anterior extremity of right spicule	expanded	**expanded**	expanded	expanded	–	–
Spicular ratio	3.37	**3.83**	4.1	3.52	3.7	± 0.3
Cuticular bosses	only ventral	**only ventral**	only ventral	only ventral	–	–
Diameter of cuticular bosses at mid-body	4	**4**	5	3	4	± 0.8
Distance between bosses of area rugosa*	10	**8**	5	6	7.3	± 2.2
Distance between bands of area rugosa*	26	**29**	19	18	23	± 5.4

* 3600 to 3900 from tail tip;

a single papilla;

b paired papillae; SD: standard deviation; ND: not determined; all measurements are in micrometres, unless otherwise stated.

**Table III.** Comparative characteristics of the females of the species of *Ochoterenella*.

*Ocbotereella* species	Authority	Body length	Body width	Oesophagus total length	Oesophagus total length	Apex to vulva	Position of vulva^a^	Tail length	Type host	Host family	Type country
*esslingeri* n. sp.	This paper	34,7–36,6 (36)	390–470 (445)	1132–1570 (1365)	885–1250 (1045)	1930–2360	intestinal	200–260	*Bokerma nnohyla luctuosa*	Hylidae	Brazil
*convoluta*	(Molin, 1858)*	27–32(29.5)	500	ND	ND	ND	ND	270	*Leptodactylus pentada ctylus*	Leptodactyl idae	Brazil
*sealaris*	(Travassos, 1929)	ND	ND	ND	ND	ND	ND	ND	*Leptodactylusocellatus*	Leptodactyl idae	Brazil
*vellardi*	(Travassos, 1929)	37–50(43.5)	ND	ND	ND	ND	ND	**1000**	Rhinellamarina***	Bufonidae	Brazil
*digiticaudata*	Caballero, 1944**	**44–57(51)**	**564–673(605)**	**1486–2474(1896)**	**1238–1589(1537)**	1020–1782(1420)	**oeso**	**371–639(456)**	*Rhinella marina*	Bufonidae	Mexico
*guyanensis*	Bain & Prod’Hon. 1974)	**47–57(52)**	260–450(356)	**1860**	1550	**1250**	**oeso**	640	*Rhinella marina*	Bufonidae	FrenchGuyana
*albareti*	(Bain *et al*, 1979)	**49 & 55**	**650 & 645**	**2465 & 2910**	**2220 & 2650**	2200	oeso	250 & 330	*Rhinella marina*	Bufonidae	FrenchGuyana
*dufourae*	(Bain *et al*, 1979)	32–44	**560**	1750	950–1600	**760–1410**	**oeso**	128–285	*Rhinella marina*	Bufonidae	FrenchGuyana
*oumari*	(Bain *et al*, 1979)	39	**590**	**2160**	**1800**	**1100**	**oeso**	280	*Rhinella marina*	Bufonidae	FrenchGuyana
*royi*	(Bain *et al*, 1979)	32–69(51)	400–520(460)	**2370**	**2070–2400**	**1020–1650**	**oeso**	240–410	*Rhinella marina*	Bufonidae	FrenchGuyana
*caballeroi*	Esslinger, 1987	**44 & 49**	416 & 436	**1832 & 1931**	**1565 & 1705**	**1104 & 1406**	**oeso**	259 & 370	*Rhinella marina*	Bufonidae	Mexico
*nanolarvata*	Esslinger, 1987	38.8–47.9(43.1)	485–594(528)	**1724–2316 (1927)**	**1436–1851 (1665)**	1197–1960	**oeso**	144–320	*Rhinella marina*	Bufonidae	Mexico
*chiapensis*	Esslinger, 1988	37.7–57.6 (48.7)	376–624 (497)	**1753–2624 (2235)**	**1535–2307 (1952)**	881–2099 (1529)	**oeso**	168–394	*Rhinella marina*	Bufonidae	Mexico
*figueroai*	Esslinger, 1988	**58–71 (65)**	**564–702 (607)**	**2811–3980 (3159)**	**2406–2792 (2652)**	1683–2574 (2141)	**oeso**	293–504(389)	*Rhinella marina*	Bufonidae	Guatemala
*lamothei*	Esslinger, 1988	**47–57 (52)**	446–594 (531)	**2149–2653 (2440)**	**1832–2297 (2081)**	1554–2277 (1944)	**oeso**	173–319	*Rhinella marina*	Bufonidae	Mexico
*complicata*	Esslinger, 1989	27–35 (30)	356–594 (465)	1188–2010 (1485)	911–1733 (1217)	**762–1273 (1013)**	**oeso**	204–281 (244)	*Rhinella marina*	Bufonidae	Columbia

* reference of description: Travassos, 1929;

** reference of description: Esslinger, 1986;

*** *Rhinella marina* (Linnaeus, 1758) = *Bufo marinus*;

a position of vulva in relation to digestive tract; figures in brackets indicate the range where available; bold: characters distinct from the present material; ND: not determined; oeso: oesophageal.

**Table IV.** Comparative characteristics of the microfilariae and cuticular bosses of the females of the species of *Ochoterenella*.

		Microfilaria				Cuticular bosses at mid-body		
		
							Distance between	
*Ochoterenella species*	References	Length	Maximum width	Anterior end	Posterior end	Length	bosses	bands
*esslingeri* n. sp.	This paper	97–132	4–4.5*	wider than mid-body	attenuated	absent**	absent**	absent**
*convoluta*	Travassos, 1929	ND	ND	ND	ND	**6–20**	**irregular**	no bands
*scalaris*	Travassos, 1929	ND	ND	ND	ND	**20**	**8**	**5–6**
*vellardi*	Travassos, 1929	ND	ND	ND	ND	**16**	**80–120**	**40–112**
*digiticaudata*	Esslinger, 1986	96–134	2.6–3.6	**as wide as mid-body**	**not attenuated, rounded tip**	**7–10**	**13–20**	**62–93**
*guyanensis*	Bain and Prod’hon, 1974	130–190	4.5	**slightlyattenuated**	**not attenuated, rounded tip**	**5**	**4–5**	**30–35**
*albareti*	Bain *et al*, 1979	**62–68**	**5–5.5**	**as wide as mid-body**	attenuated	**20**	**30–120**	**18–20**
dufourae	Bain *et al*, 1979	108–138	4	**as wide as mid-body**	**slightly attenuated**	**4–7**	**10–20**	**30–80**
*oumari*	Bain *et al*, 1979	88–99	5	**slightly attenuated**	attenuated	**6–12**	**10–40**	**40–50**
*royi*	Bain *et al*, 1979	130–163	5	wider than mid-body	attenuated	**7–15**	**7–15**	**30–50**
*caballeroi*	Esslinger, 1987	**76–88**	3.4–4.1	**subterminal constriction**	attenuated	**9–16**	**15–48**	**36–69**
*nanolarvata*	Esslinger, 1987	**51–67**	5.1–6.2*	wider than mid-body	**constricted**	**8–15**	**28–37**	**35–44**
*chiapensis*	Esslinger, 1988	**68–91 (80)**	**5–7 (6.4)**	**sligthly constricted**	**abruptly attenuated**	**10–15**	**20–70**	**30–40**
*figueroai*	Esslinger, 1988	**74–85 (81)**	5.3–6.3 (5.7)*	wider than mid-body	attenuated	**10–24**	**37–44**	**58–67**
**lamothei**	Esslinger, 1988	88–96 (91)	5.4–6.2*	wider than mid-body	attenuated	**7–18**	**28–42**	**48–59**
*complicata*	Esslinger, 1989	96-114 (104)	3.6–4.4	**as wide as mid-body**	**not attenuated, rounded tip**	**3–7**	**18–27**	**26–37**

* maximum width near anterior end;

** present in caudal region, rounded, 3-6 in diameter, irregularly arranged; figures in brackets indicate the range where available; bold: characters distinct from the present material; ND: not determined.

**Table V.** Comparative characteristics of the males of the species of *Ochoterenella*.

*Ochoterenella* species	Reference	Bodylength	Body width	Oesophagus total length	Glandular oesophagus length	Taillength	Tail width	Spicule lengthLeft Right		Caudal PapillaePrecloacal Postcloacal		Caudal alae
*esslingeri* n. sp.	This paper	14,9–19.1	190–280	890–1160	640–840	160–203	70–118	320–345	91-135*	l^a^ + 2^b^	2 + 2 + 2	absent
*convoluta*	Travassos, 1929	ND	ND	ND	ND	**72**	ND	346–375	144–150	2 + 2	2 + 2 + 2	**thin**
*scalaris*	Travassos, 1929	ND	ND	ND	ND	ND	ND	230–350	130–150	2	2 + 2 + 2	absent
*vellardi*	Travassos, 1929	36	ND	ND	ND	ND	ND	**250–270**	**160–170**	**2 + 2**	2 + 2 + 2	**thin**
*digiticaudata*	Esslinger, 1986	16.5–24.1	267–366	1287–1832	**1138–1584**	117–180	87–116	**167–240**	119–146	1 + 2	[2 + 2] + 2	absent
*guya nensis*	Bain and Prod’hon, 1974	**21 & 25**	**370**	**1885**	**1625**	157	72	**193**	121	1 + 2	2 + 2 + 2	**thin**
*oumari*	Bain et al., 1979	**22.7**	305	**1900**	**1600**	115	65	**168**	100	1 + 2	2** + 2 + 2	absent
*royi*	Bain *et al.*, 1979	**26 & 27**	**400**	**2020**	**1755**	135 & 165	100	**245 & 280**	130 &140	1 + 2	2 + 2 + 2	absent
*figueroai*	Esslinger, 1988a	**22–27**	276–376	**1796–2604**	1544–2129	132–170	84–125	**178–243**	120–147	1 + 2	[2 + 2] + 2	absent
		**(24)**	(322)	**(2199)**	**(1842)**	(154)	(101)	**(204)**	(133)			

* large capitulum;

** small size contrasting with other pairs in *O.oumari*;

a single papilla;

b paired papillae; [] pairs close together; figures in brackets indicate the range where available; bold: characters distinct from the present material; ND: not determined.

## Results

### *Ochoterenella Esslingeri* N. Sp. Souza Lima & Bain

**Fig. 1.**
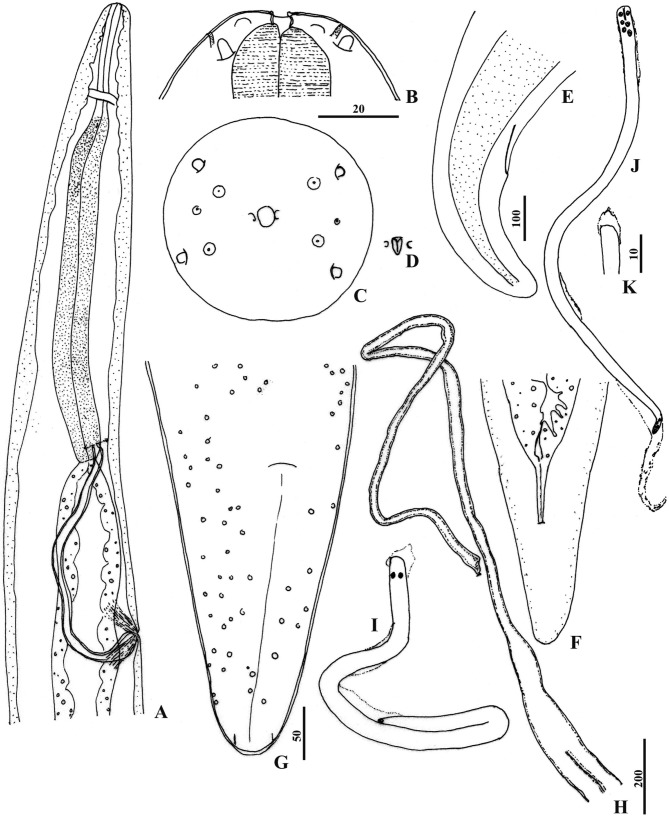
*Ochoterenella esslingeri* n. sp. A-H: Female. A, anterior region, right lateral view (coils of ovijector not represented); B, head, dorso-ventral view (holotype); C, head, apical view; D, buccal capsule, optical transverse section; E, tail, right lateral view, lateral chord dotted (holotype); F, tail, ventral view; G, cuticular bossesin the caudal region, ventral view; H, ovijector and beginning of the uteri, after dissection. I-K: Microfilaria. I, immature folded microfilaria,extracted from uteri; J, mature microfilaria from uteri within sheath with small, refractile granules; K, head with small hook and sheath. Scales in μm: A, H, 200; B, C, I, J, 20; D, K, 10; E, F, 100; G, 50.

**Fig. 2.**
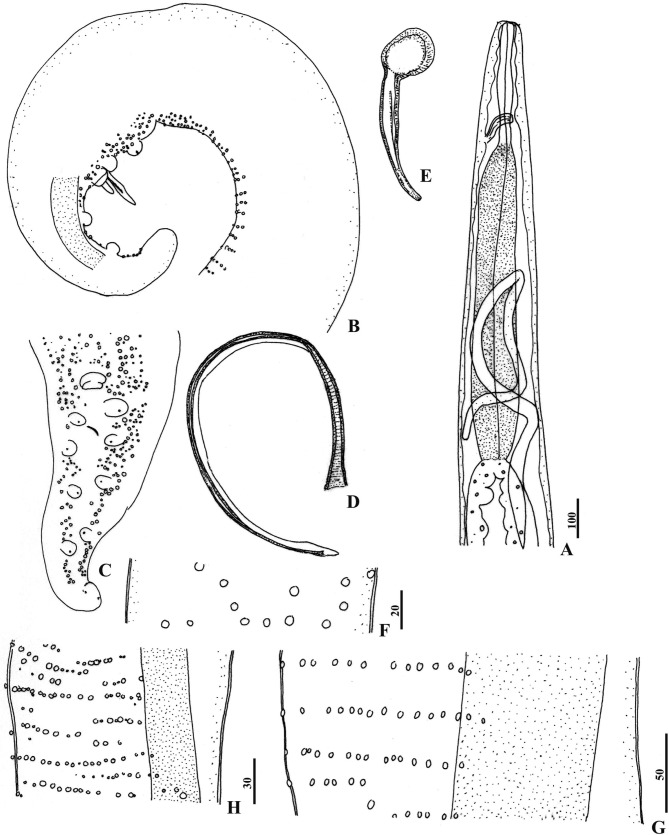
*Ochoterenella esslingeri* n. sp., male. A, anterior region, left lateral view; B, caudal region, lateral view (lateral chord dotted); C, tail, ventral view; D, left spicule, right lateralview; E, right spicule, right lateral view; F, cuticular bosses irregularly arranged in anterior region, dorso-ventral view; G, bands of cuticular bosses in anterior third region, 5,200 from apex, left lateral view (lateral chord dotted); H, cuticular bosses of the *area rugosa*, 1,250 from tail tip, left lateral view (lateral chord dotted). Scales in μm: A-C, 100; D, E, G, 50; F, 20; H, 30.

The description is based on seven females and four males (Figs 1, 2 ; Tables I, II).

#### • Female

Body cylindrical, anterior and posterior extremities gradually attenuated (Fig. 1A, E), maximum body width in the vulvar region. Cuticle thin, without lateral alae. Cuticular ornamentation present only in the caudal region (Fig. 1G); bosses on ventral and dorsal aspect, small and rounded, three-six in diameter, irregularly arranged and varying in density between females. Width of lateral chords about half of body width at mid-body. Head rounded, with flattened top. Rectangular cephalic plate expanded laterally, 53–58 × 30–36 (Fig. 1B, C), with two pairs of external labial papillae and two pairs of cephalic papillae, each with a prominent cuticularized process (“articulated papillae”, Bain & Prod’Hon, 1974); amphids small. Circular mouth; a pair of small lateral cuticular flap-like parastomal structures. Buccal capsule small and weakly cuticularized; buccal cavity 3 long and 6 wide, its lumen Y-shaped in transverse section (Fig. 1B, D). Oesophagus divided into short anterior muscular portion and long, thick glandular portion (Fig. 1A); oesophagus ratio 3.2–4.3. Intestine broad with wide lumen. Vulva, a transverse slit, posterior to oesophago-intestinal junction; radiating muscles attached to vulva and directed laterally from its opening; vulvar ratio 4.8–6.5. No vagina differentiated; ovijector 2,920 long, simple (Fig. 1H), extending anteriorly, bifurcated to form uteri after coiling around glandular oesophagus; amphidelphic. Anus on a small elevation, tail conical, extremity rounded (Fig. 1E, F); phasmids identified; tail ratio 1.9–2.8.

#### • Male

Anatomy of head and oesophagus as in female, but processes of cephalic papillae shorter. Laterally elongated rectangular cephalic plate 54 × 30. Posterior region helically coiled with three-five turns (Fig. 2B). Rounded cuticular bosses present on the ventral surface of the body from the glandular oesophageal region to the caudal region; bosses initially large, not numerous and irregularly arranged (Fig. 2F), becoming more numerous and organized along the body, to gradually form transverse bands; distance between bosses within a band, measured at 3,700–3,900 from tail tip, about three times shorter than between bands (Fig. 2G). In the *area rugosa*, the start of which is indistinguishable from the ventral ornamentation of the body, the bosses are smaller and more numerous (Fig. 2H). In the caudal region, the *area rugosa* is made of smaller bosses and again irregularly arranged (Fig. 2C). Caudal papillae: a single large precloacal papilla (or plaque, according to Esslinger, 1986a, 1987, 1988) with an internal transverse furrow, and four pairs of large sessile papillae; the latter arranged symmetrically in two groups: one precloacal pair; three postcloacal pairs, equidistant (20 to 30 apart), the last pair located about 50 from end of tail (Fig. 2B, C). Spicules distinctly unequal and dissimilar, spicular ratio 3.7–4.1; right spicule simple, distal end tapered and rounded, proximal end expanded and strongly cuticularized for the insertion of the retractor muscle (Fig. 2E); left spicule slender, ventrally curved, with narrowing at the transition between handle and blade; blade about two thirds of the spicule length, lined with narrow alae, slightly widening distally; attennuated membranous tip (Fig. 2D).

#### • Microfilariae

Sheath present, exceeding the length of the larva to a larger or lesser extent at the anterior and posterior extremities (Fig. 1J); tiny refractile granules seen along its entire length. At dissection, microfilariae adhered to each other and to uterine wall. Anterior extremity wider and rounded, body gradually tapering to posterior region; very small cephalic hook (Fig. 1K); short cephalic space, oesophageal axis often conspicuous in anterior end (Fig. 1J); rounded tail tip with terminal nucleus (Fig. 1I, J). Measurements (n = 25, from paratype): body 112 ± 24 (97–132) long, 4.5 ± 0.7 (4–6) wide; cephalic space 2.5 long. Immature microfilariae folded in sheath (Fig. 1I).

Type host: *Bokermannohyla luctuosa* (Pombal & Hadad, 1993) (Anura: Hylidae), a single type host specimen deposited in “Coleção Herpetologia/Anfíbios, Departamento de Zoologia, Universidade Federal de Juiz de Fora”, registration number 968.

Type locality: Parque Municipal da Lajinha (21º 47’ 34.14’’ S – 43º 22’ 03.28’’ W), Juiz de Fora, Minas Gerais, Brazil.

Type material: female holotype, male allotype, eight female and seven male paratypes (172 YU); deposited in the helminth collection of the Muséum National d’Histoire Naturelle (MNHN), Paris. Other paratypes deposited in the Laboratório de Taxonomia e Ecologia de Helmintos, Departamento de Zoologia, Instituto de Ciências Biológicas, Universidade Federal de Juiz de Fora, Brazil (accession number B8–13).

Site of infection: body cavity and muscular aponeuroses of the thighs.

Prevalence and intensity: a single host specimen with 24 male and 32 female nematodes.

Etymology: named in honor of J.H. Esslinger for his contribution to the knowledge of the biodiversity of Neotropical Waltonellinae and other filarial nematodes.

## Taxonomic Discussion

The filariae described in this paper present the main characters of the genus *Ochoterenella* as redefined by Esslinger (1986a, b): cuticularized parastomal structures, distinct buccal capsule, no lateral or caudal alae. The single discrepancy found is that the “bands of longitudinally oriented bosses in mid-region” which, according to Esslinger (1986a, b), are present in both sexes, are absent in the current females. However, bosses were not entirely absent in the females studied by us, but they were restricted to the posterior region; they are rounded and irregularly arranged. Using the key proposed by Esslinger (1989) for *Ochoterenella*, which is mainly based on the cuticular ornamentation of females, as males are often unknown, the studied specimens are clearly different from the 15 species described to date.

Numerous other characters distinguish the present material from the remaining species of *Ochoterenella* (Tables III-V). In ten species the glandular oesophagus is longer, nearly reaching or just exceeding 2,000; in descending order of length, these species are *O. figueroai*, *O. albareti*, *O. royi*, *O. chiapensis*, *O. lamothei*, *O. oumari*, *O. nanolarvata*, *O. caballeroi*, *O. digiticaudata* and *O. guyanensis* (Bain & Prod’Hon, 1974; Bain *et al.*, 1979; Esslinger, 1987, 1988a, b). Among these species, the tail of the female is longer and the microfilariae are cylindrical with a rounded tail tip in *O. digiticaudata* and *O. guyanensis*; the microfilariae are shorter in *O. albareti*, *O. caballeroi*, *O. nanolarvata* and *O. chiapensis*, and they are also distinct in having an attenuated tail, with even an abrupt constriction in the last species. The males of *O. figueroai, O. royi*, *O. oumari*, *O. digiticaudata* and *O. guyanensis* have a shorter left spicule (≤ 280), particularly *O. oumari* (168).

Considering the two species in which the glandular oesophagus is similar to the present specimens, females of *O. dufourae* are distinct in having a short robust tail (Bain *et al.*, 1979), and *O. complicata* has microfilariae in which the posterior region is not attenuated and has a rounded tip (Esslinger, 1989).

The oesophagus was not measured in the remaining three species, *O. convoluta*, *O. scalaris* and *O. vellardi*, but detailed descriptions of their cuticular ornamentation, in which they are distinct from the present material, were provided. No illustrations but some measurements (Travassos, 1929) are available for the following two species: *O. vellardi* females have a long tail (1,000), and males have a shorter left and longer right spicule; two precloacal pairs of papillae are reported, as also in *O. convoluta*, but this might be an erroneous interpretation, the unpaired papilla being as large and salient as the paired papillae.

The single species of *Paraochoterenella* must be considered as well, since the definition of the genus does not appear clearly distinct from that of *Ochoterenella* when comparing Esslinger (1986b) and Purnomo & Bangs (1999). *Paraochoterenella javanensis* Purnomo & Bangs, 1999, a parasite of the dicroglossid *Fejervarya cancrivora* (Gravenhorst, 1829) (= *Rana cancrivora*) in Indonesia, was described as “cuticular bosses minute (< 2–3), non bacillary in appearance, with irregular distribution”. Therefore it is rather similar to the present material, but the bosses are not restricted to the posterior region. Moreover, in *P. javanensis*, both sexes are smaller, and the male differs in the absence of an unpaired precoloacal papilla. In addition, the male is distinct in having two precloacal and four postcloacal pairs of papillae (instead of one and three, respectively), and the *area rugosa* is organized in transverse bands anterior and posterior to the cloacal aperture.

We therefore conclude that the material described herein represents a new species, *Ochoterenella esslingeri* n. sp.

## Discussion

*Ochoterenella esslingeri* n. sp. expands the host range of the genus to the Hylidae. Some representatives of this anuran family have been listed as hosts of a few *Ochoterenella* species that were described from other type hosts (Vicente *et al.*, 1990; Azevedo-Ramos *et al.*, 1998; Goldberg & Bursey, 2008). However, in cases where filarial identifications were not based on detailed morphological studies, these data ought to be considered with caution, since the works of Esslinger (1986a, 1987, 1988) demonstrated that worms identified as *O. digiticaudata* in the collection of Prof. E. Caballero in the Instituto de Biologia at Universidad Nacional de Mexico, contained three hidden species, *O. caballeroi*, *O. nanolarvata* and *O. chiapensis*.

*Ochoterenella esslingeri* n. sp. presents the main generic characters of *Ochoterenella*, and the slight particularities that were seen in the new species (female ornamentation and position of the vulva) do not deserve a higher taxomic rank than specific. The two species parasitic in Leptodactylidae are too poorly known to draw any conclusions. What remains is an incredibly high diversity of *Ochoterenella* in the giant toad, *R. marina* (Travassos, 1929; Caballero, 1944; Bain & Prod’Hon, 1974; Bain *et al.*, 1979; Esslinger 1986a, 1987, 1988a, b, 1989). This poses the question of the origin of this diversity. The geographic range of the giant toad is large, extending from Colombia to Brazil in South America, to Guatemala and Mexico in Central America. The vectors of these filariae are culicids (see review in Bain & Chabaud, 1986). Either diversification might have occurred from a single ancestral species, but this should be supported by some distinctive traits, or the giant toad is parasitized by species from co-occurring anuran hosts in the surrounding environment, or a mixed evolutionary process took place.

*Paraochoterenella*, although not clearly different from *Ochoterenella* in the original definition, very likely represents a distinct genus with the main character being its caudal papillae: the two pairs of precloacal papillae, distinctly anterior to the cloacal aperture as stressed by Purnomo & Bangs (1999), and the absence of an unpaired papilla. In addition, the *area rugosa* forms transverse bands near the cloacal aperture. Finally, the cuticular bosses of *P. javanensis* are strangely drawn and do not seem to be salient (Purnomo & Bangs, 1999, Figs 11–17). In contrast, the absence of a sheath in the microfilaria is not decisive because this delicate character is often very difficult to observe, particularly in Giemsa stained blood smears, where the sheath often remains unstained. It is expected that more species will be described in the Oriental Realm and will support this interpretation of a particular lineage of Waltonellinae. The present references on Waltonelinae from this region do not allow a generic assignation (Johnston, 1967; Moravec & Sey, 1985), except that of Petit & Yen (1979) in Malaysia, but it concerns a species of *Foleyellides* according to Esslinger (1986b). Interesting materials from anurans were reported more recently in India (Sarkar & Manna, 2008; Oinam & Gambbir, 2011), but descriptions were not accurate and the generic assignation to *Ochoterenella* was not supported.
